# Reshaping the Cone-Mosaic in a Rat Model of Retinitis Pigmentosa: Modulatory Role of ZO-1 Expression in DL-Alpha-Aminoadipic Acid Reshaping

**DOI:** 10.1371/journal.pone.0151668

**Published:** 2016-03-15

**Authors:** Wan-Qing Yu, Yun Sung Eom, Jung-A Shin, Divya Nair, Sara X. Z. Grzywacz, Norberto M. Grzywacz, Cheryl Mae Craft, Eun-Jin Lee

**Affiliations:** 1 Neuroscience Graduate Program, University of Southern California, Los Angeles, California, United States of America; 2 Dornsife College of Letters, Arts and Sciences, University of Southern California, Los Angeles, California, United States of America; 3 Mary D. Allen Laboratory for Vision Research, USC Eye Institute, University of Southern California, Los Angeles, California, United States of America; 4 Department of Biomedical Engineering, Viterbi School of Engineering, University of Southern California, Los Angeles, California, United States of America; 5 Department of Ophthalmology, Keck School of Medicine, University of Southern California, Los Angeles, California, United States of America; 6 Department of Cell & Neurobiology, Keck School of Medicine, University of Southern California, Los Angeles, California, United States of America; 7 Department of Anatomy, School of Medicine, Ewha Womans University, Seoul, Korea; 8 Department of Neuroscience, Georgetown University, Washington D.C., United States of America; 9 Department of Physics, Georgetown University, Washington D.C., United States of America; 10 Department of Biological Structure, University of Washington, Seattle, United States of America; National Eye Institute, UNITED STATES

## Abstract

In S334ter-line-3 rat model of Retinitis Pigmentosa (RP), rod cell death induces the rearrangement of cones into mosaics of rings while the fibrotic processes of Müller cells remodel to fill the center of the rings. In contrast, previous work established that DL-alpha-aminoadipic-acid (AAA), a compound that transiently blocks Müller cell metabolism, abolishes these highly structured cone rings. Simultaneously, adherens-junction associated protein, Zonula occludens-1 (ZO-1) expression forms in a network between the photoreceptor segments and Müller cells processes. Thus, we hypothesized that AAA treatment alters the cone mosaic rings by disrupting the distal sealing formed by these fibrotic processes, either directly or indirectly, by down regulating the expression of ZO-1. Therefore, we examined these processes and ZO-1 expression at the outer retina after intravitreal injection of AAA and observed that AAA treatment transiently disrupts the distal glial sealing in RP retina, plus induces cones in rings to become more homogeneous. Moreover, ZO-1 expression is actively suppressed after 3 days of AAA treatment, which coincided with cone ring disruption. Similar modifications of glial sealing and cone distribution were observed after injection of siRNA to inhibit ZO-1 expression. These findings support our hypothesis and provide additional information about the critical role played by ZO-1 in glial sealing and shaping the ring mosaic in RP retina. These studies represent important advancements in the understanding of retinal degeneration’s etiology and pathophysiology.

## Introduction

Retinitis Pigmentosa (RP) is characterized by an initial loss of rod photoreceptors, followed by a slow, progressive loss of cones and rewiring of the remaining retinal neurons [1, 2]. In addition, Müller cells increase intermediate filament synthesis and form a dense fibrotic seal encompassing the outer retina [1, 3]. Whenever the photoreceptors are depleted, the formation of a distal glial seal is common in retinal degenerations in both animals [1] and humans [3].

In RP rhodopsin S334ter-line-3 rat, death of rods induces cones to lay flat against the outer retina, which is observed in vertical sections. There are distinct regions full of clusters of cell bodies of cone that are adjacent to regions devoid of cell bodies but are richer in long processes [4, 5]. At the same time, Müller cells processes form a dense fibrotic seal or barrier in the outer retina [4, 6]. In whole-mount retina, cones are distributed in an orderly mosaic of rings. Müller cell processes cluster in broccoli-like shapes to occupy these zones, interact with the cones, and induce cone migration to the edges of the holes of rods [7, 8]. Furthermore, glial fibrillary acidic protein (GFAP) expression appears in processes of Müller cells filled in cone rings [7].

Previously, we tested if Müller cell processes are necessary and sufficient for the rings to exist. To test the relevance of this interaction, we injected a drug known to disrupt Müller cell metabolism, DL-α-aminoadipic acid (AAA). The disruption of cone rings suggested that the maintenance of these cone rings in the RP is dependent on the close interactions with Müller cells [7]. Furthermore, intravitreal injection of AAA in mice transiently disrupts the integrity of the outer limiting membrane (OLM) [9]. At the OLM, apical processes of Müller cells and inner segments of rods and cones are joined together with a specialized adherens junction associated protein, ZO-1 [10–14]. In the RP retina, ZO-1 expression is associated with the network of rings of cones [7]. Thus, we hypothesized that AAA treatment disrupts cone rings by attacking the distal sealing formed by the fibrotic processes of Müller cells. Subsequently, either directly or indirectly, ZO-1 down-regulation is triggered between the Müller cells and cones. In this study, we further investigated the fibrotic processes of Müller cells and the expression of ZO-1 in the outer retina after intravitreal injection of AAA and defined ZO-1’s contribution using molecular tools in this intricate process with siRNA inhibition of ZO-1 expression.

## Materials and Methods

### Animals

The third line of albino Sprague-Dawley rats homozygous for the truncated murine opsin gene (creating a stop codon at Serine residue 334; S334ter-line-3) was generously provided for our studies from Matthew LaVail (University of California, San Francisco, CA, USA). Homozygous S334ter-3 male rats were mated with homozygous S334ter-3 female rats to produce offspring for the S334ter-3 transgene used for this study and referred to as the RP model in this paper. RP rats were euthanized at postnatal (P) days 30, 31, 32, 33, 37, 44, 50, and 51 (N = 10 for each stage). Controls were Sprague-Dawley rats euthanized at P50 (N = 5; Harlan, Indianapolis, IN). Both sexes of control and RP rats were used. All rats were housed under cyclic 12:12 hour (hr) light/dark conditions with free access to food and water. The University of Southern California’s Institutional Animal Care and Use Committee approved the research protocol for use of rats in this study. Research was conducted in adherence with the Association for Research in Vision and Ophthalmology (ARVO) Statement for the Use of Animals in Ophthalmic and Visual Research. All surgeries on rats were performed under anesthesia induced by intra-peritoneal injection of ketamine (100 mg/kg; KETASET, Fort Dodge, IA) and xylazine (20 mg/kg; X-Ject SA, Butler, Dublin, OH) or Euthasol (40 mg/kg body weight).

### Administration of Alpha-aminoadipic Acid (AAA)

DL-α-Aminoadipic acid (AAA, Sigma-Aldrich Corp., St. Louis, MO, USA) was prepared in phosphate-buffered saline (PBS), adjusted to pH 7.5 and sterile-filtered before administration. AAA was administered by intravitreal injection with a fine-glass microelectrode through the sclera at the level of the temporal peripheral retina. Higher concentrations of AAA are known to affect the metabolic pathway in photoreceptors [9, 15]. For preliminary testing, 4 μl of three concentrations of the AAA (5, 10, 50μg/ml) were intravitreally injected into the eyes of RP rats at P30. Survival periods of 1–3 days, 1 week and 2 weeks were tested. The 5μg/ml of AAA did not disrupt cone rings in RP retina; however, 10μg/ml and 50μg/ml of AAA gave similar end results in terms of the degree of change in the distribution of cones. Thus, 10μg/ml of AAA was selected. The optimal stage for the injection of AAA was P30 when cones were arranged in rings across the entire retina. The cone rings were disrupted after 3 days of AAA post-injection. Thus, as for survival periods, 3 days and 2 weeks were used. Sham injections, for control, consisted of 4μl sterile saline. For each animal, one eye was injected with AAA and the other eye was used to inject saline for comparison. Surgeries on rats were performed under anesthesia induced by intra-peritoneal injection of ketamine (100 mg/kg; KETASET, Fort Dodge, IA, USA) and xylazine (20 mg/kg; X-Ject SA, Butler, Dublin, OH, USA). The entire injection procedure required only a few minutes, which allowed us to complete the injection before the animals recovered from anesthesia. Following surgeries, veterinary ophthalmic antibacterial ointment was applied to prevent drying of cornea and infection.

### Preparation and Administration of siRNA

The siRNA technology to produce a knockdown of target proteins in the eye is well established [12, 16]. We targeted siRNA against ZO-1 expression by generating pre-designed silencer select ZO-1 siRNA (Gene Name: tight junction protein 1; Gene Aliases: ZO-1; Locus ID: 292994, Species: Rat, siRNA ID: s146925, Invitrogen, Carlsbad, CA, USA). The siRNA was resuspended and diluted to the appropriate concentration in sterile buffer containing lipofectamine using RNAase-free plasticware. Controls have a 4μl injection of Silencer Select Negative Control siRNA (Catalog# 4390843, Invitrogen). ZO-1 siRNAs were applied by intravitreal injection to the temporal peripheral retina at P50. Two concentrations of ZO-1 siRNA (15 and 25μM) were administered and monitored after 24 hr and 48 hr. However, 15 μM of ZO-1 siRNA did not disrupt the cone ring and 48 hr of ZO-1siRNA reduced the cone number. Thus, ZO-1 siRNA of 25μM was used for 24 hr. This was determined experimentally as the most efficient in terms of the degree of change in the mosaics of M-opsin cones.

### Tissue Preparation

Animals were deeply anesthetized by intra-peritoneal injection of Euthasol (40 mg/kg body weight, Virbac, Fort worth, TX, USA) and the eyes were enucleated. Animals were then euthanized by an overdose of Euthasol. For secondary method we will perform thoracotomy or decapitations. Their eyes’ anterior segments were then removed and the eyecups were fixed by immersion in 4% paraformaldehyde in 0.1 M phosphate buffer (PB), pH 7.4, for 1.5–2 hr. Following fixation, the retinas were carefully dissected and transferred to 30% sucrose in PB for 24 hr at 4°C. For storage, all retinas were then frozen in liquid nitrogen, stored at -70°C, thawed, and rinsed in 0.01 M phosphate buffered saline (PBS; pH 7.4). For cryostat sections, eyecups were embedded in OCT embedding medium (Tissue-Tek, Elkhart, IN, USA), then quickly frozen in liquid nitrogen and subsequently sectioned along the vertical meridian on a cryostat at a thickness of 20 μm.

### Immunohistochemistry

For fluorescence immunocytochemistry, 20μm thick cryostat sections were incubated in 10% normal goat serum (NGS) or 10% normal donkey serum (NDS) and 1% Triton X-100 in PBS for 1 hr at room temperature. Sections were then incubated overnight with a rabbit polyclonal antibody directed against: mouse green opsin (M-opsin, dilution 1:1000) [17] and glial fibrillary acidic protein (GFAP, Sigma; 1:500); mouse monoclonal antibody directed against glutamine synthetase (GS, Millipore Temecula, CA, USA; 1:1000), β-tubulin (Sigma; dilution 1:500) and zonula occludens (ZO-1, Invitrogen; 1:500). Each antiserum was diluted with PBS containing 0.5% Triton X-100 at 4°C. Retinas were washed in PBS for 45 min (3 × 15 min). Afterwards, the retinas were incubated for 2 hr in carboxymethylindocyanine (Cy3)-conjugated affinity-purified, donkey anti-rabbit IgG (Jackson Immuno Labs, West Grove, PA, USA; dilution 1:500); Alexa 488 anti-mouse (Molecular Probes, Eugene, OR, USA, dilution 1:300) or Cy5-conjugated, donkey anti-mouse IgG (Jackson Immuno Labs; dilution 1:300) at room temperature. The sections were washed for 30 min with 0.1M PBS and cover slipped with Vectashield mounting medium (Vector Labs, Burlingame, CA, USA). For whole-mount immunohistochemical staining, the same procedure was used. For M-opsin and GS, the primary antibody incubation was for 2 days and the secondary antibody incubation was for 1 day. FITC-conjugated mouse monoclonal antibody directed against ZO-1 (Invitrogen; dilution 1:500) was incubated for 1 day.

For double and triple labeling, sections and whole mounts were incubated in a mixture of following primary antibodies: GS and ZO-1; ZO-1, GS, and M-opsin, followed by the appropriate secondary antibodies and processed. Sections and whole-mounts were then analyzed using a Zeiss LSM 510, (Zeiss, NY, USA) confocal microscope. Immunofluorescence images were processed with Zeiss LSM-PC software. The brightness and contrast of the images were adjusted using Adobe Photoshop 7.0 (Adobe Systems, San Jose, CA, USA). For presentation, all Photoshop manipulations for brightness and contrast only were carried out equally across all sections.

### Statistical analysis

The density of M-opsin cones was counted in three retinal whole-mount preparations from each group. In RP and AAA-treated RP retinas, due to loss of some cone outer segments (COS), we counted their cell bodies. Loss of COS was previously detected in *rd1*^*-/-*^ mice [18, 19] and S334ter-line-3 rats [20, 21]. Confocal micrographs of the retinas were taken at the focal level of the nuclei of M-opsin cones, covering 1x1 mm^2^ areas at the central region (1mm away from optic disc) of the superior part of the retina. At these locations we made serial optical sections using a confocal microscope. By following each M-opsin cone throughout the sections, we ensured that every M-opsin cone in the selected region was counted. Each M-opsin stained cell was marked with a white dot using the paint tool in Photoshop. Applying white dot allowed easy identification of the position of each M-opsin positive cell in the retinal area. Also, using these images, Voronoi domain and the coefficient of clustering was measured.

For the Voronoi analysis, the Voronoi domain for each cell was generated and the areas of each polygon were calculated and plotted in a histogram. To remove the artifacts induced by the edge effect, we did not include cells around the boundaries. The skewness of the Voronoi distribution was also determined. The formula used for quantifying skewness was:

$$g_{1}=\;\;\frac{\frac{1}{n}\;\sum_{i=1}^{n}{\left(x_{i}-\;\bar{x}\right)}^{3}}{(\frac{1}{n}\;\sum_{i=1}^{n}{\left(x_{i}-\;\bar{x}\right)}^{2}){\;}^{3/2}}$$

where *x*_*i*_ is the area of the *i*^*th*^ Voronoi domain and $\overset{-}{x}$ is the sample mean. Also, the coefficient of clustering (CC) is determined by the ratio between global coefficient of variance and average local coefficient of variance in Voronoi domain sizes. The formula is as follows:

$$c=\;\;\frac{n\bar{x}}{\sigma_{x}\sum_{i=1}^{n}\frac{\bar{a_{i}}}{\sigma_{a_{i}}}}$$

where *σ*_*x*_ is the standard deviation of all the Voronoi domains, ${\bar{a}}_{i}$ and $\sigma_{a_{i}}$ are the mean and the standard deviation of the size of neighboring Voronoi domains of *i*^*th*^ domain, respectively.

All the statistics were expressed as *mean* ± *SEM* (standard errors of the mean). ‘Student’s t-test was used to examine the differences among the group of means. The tests were performed and graphs were generated by MATLAB version 8.2.0 (The MathWorks Inc., Natick, MA, USA). A difference between the means of separate experimental conditions was considered statistically significant at alpha (α) level of 0.05.

## Results

### Disturbance of the cone rings in RP retinas with AAA treatment

**Fig 1:**
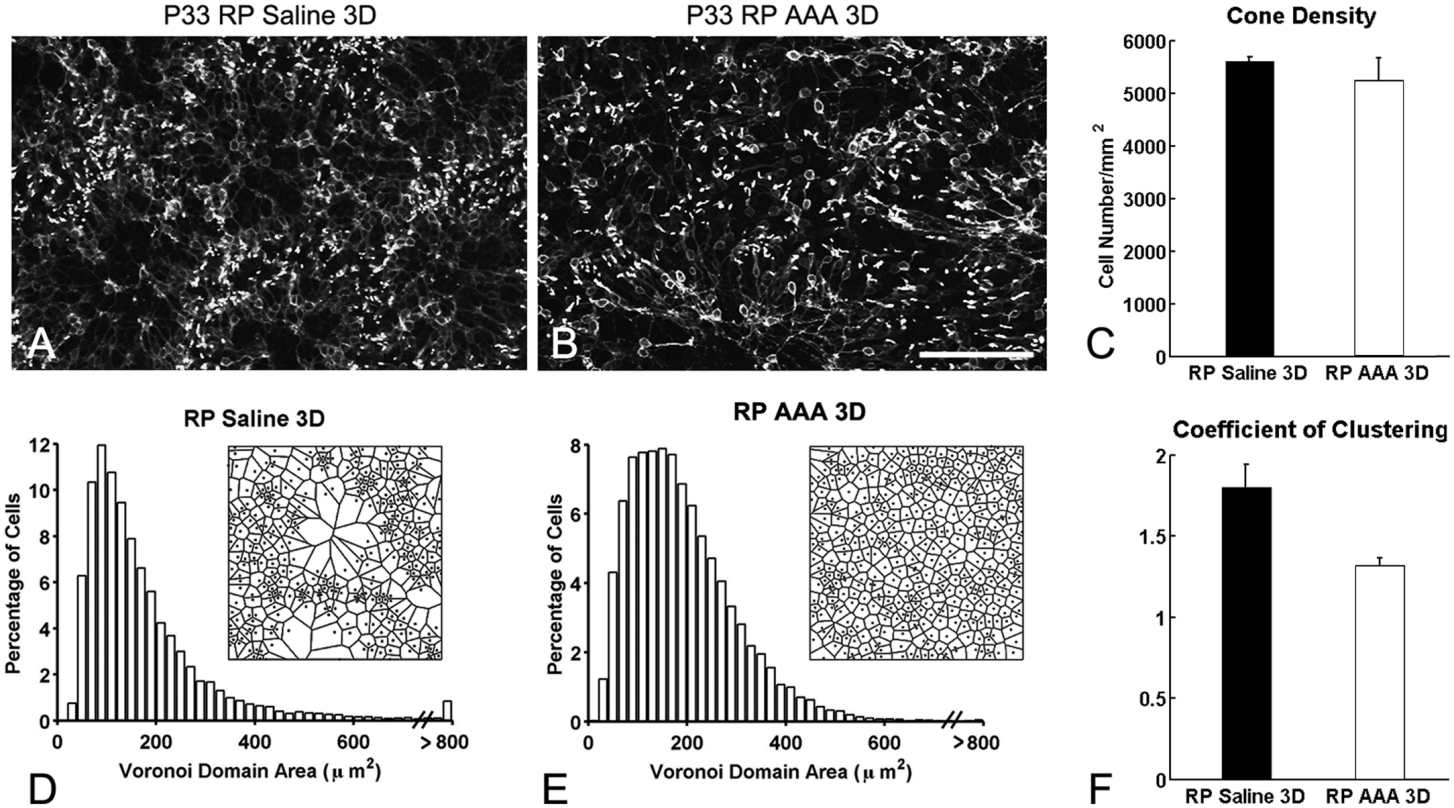
Disturbance of the cone rings in RP retinas with AAA treatment. Confocal micrographs of whole-mounts processed for M-opsin immunohistochemical staining in saline-treated RP (A), and AAA-treated (B) RP eyes. Saline and AAA (10ug/ml) were injected at P30. AAA-treated RP retinas show disruption of M-opsin rings after 3 days of injection. The summary graph illustrates mean cone density (C) measured from the 1x1 mm^2^ sampling areas (for details, see methods) of saline-treated RP (RP) and AAA-treated RP (RP AAA 3D) retinas (n = 3 animals per group). The density of M-opsin cones in AAA-treated RP retinas after 3 days showed no significant difference from RP control retinas. Histograms generated from the Voronoi analysis on the 1x1 mm^2^ sampling areas from saline-treated (D) and AAA-treated RP (E) retinas. Results are shown with survival times of 3D. Examples (~ 170 μm x 170 μm) of the resulting Voronoi domains are shown for each group (D, E). The summary graphs for the mean skewness values obtained from the Voronoi domain distribution curves are plotted for each group (D, E). Also, the graphs for the mean coefficient of clustering measured in all groups are illustrated (F). Data are presented as mean ± standard error. The symbol * indicates p < 0.05. AAA, DL-α-aminoadipic acid; P, postnatal; D, day; RP, Retinitis Pigmentosa, Scale bar = 100 μm.

Previously, we demonstrated that the specific glial-cell toxin DL-α aminoadipic acid (AAA) disrupted Müller cells in S334ter-line-3 RP retina [7]. Consistent with our previous results, we observed ring-like pattern of cones in saline-treated RP retina (control, Fig 1A). In contrast, we observed disruption of cone rings after 3 days of AAA post-injection in RP retina (Fig 1B). Together, these results suggest that the interactions between cones and Müller cells processes are necessary for the maintenance of the rings in the RP retina [7]. However, we cannot eliminate the possibility that the disruption of the cone rings is due to cone death rather than to weakening of Müller glial processes after AAA treatment. With higher concentration of AAA, photoreceptors are affected [9, 15]. Thus, we quantified the cone cell number of the M-opsin immunological stained cones within the 1x1 mm^2^ retinal areas. In P33 RP control retina, the mean density of cells was 5,600 ± 78 cells/mm^2^ compared to 5,245 ± 430 cells/mm^2^ in AAA-treated P33 RP retina, which was not significantly different (p = 0.463, Fig 1C).

Next, we examined the distribution pattern of cones using Voronoi analysis [22]. An example of Voronoi tessellation is shown in the inset besides the histogram for each group (Fig 1D and 1E). The Voronoi diagram for P33 RP control retina revealed the alternation between small and large Voronoi domains. This alternation was not random. The smaller domains were clustered with other smaller domains, while larger domains were clustered with other larger domains (Fig 1D). We quantified the degree of clustering using coefficient of clustering (CC). The CC is the ratio between the global coefficient of variation and the average local coefficient of variation in Voronoi-domain sizes [22]. The large CC indicates higher clustering. P33 RP control retinas exhibited high CC (1.80 ± 0.14), confirming that the spatial alternation between the smaller and larger Voronoi domains was not random. In contrast, in AAA-treated RP retinas, the rings disappeared and cones redistributed themselves more homogeneously. The cones appear to spread out to occupy areas inside the rings and larger Voronoi domains became smaller (Fig 1E). Thus, the distribution of Voronoi domains became more random. Therefore, the CC (1.32 ± 0.04) for AAA-treated retinas was significantly smaller than controlled group (Fig 1F, p = 0.03). This indicated cones in RP retinas became more homogeneous with AAA after 3 days. Our results indicate that AAA disrupt cone rings and redistribute cones more homogenously without a significant loss of cones.

### AAA transiently disrupts the distal glial sealing in the RP retina

**Fig 2:**
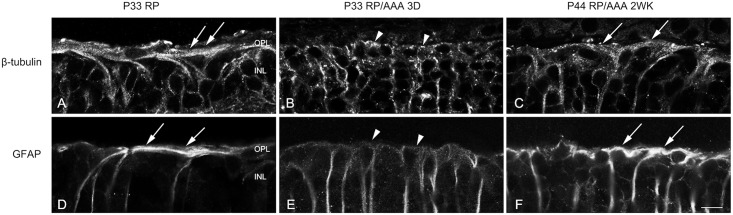
AAA transiently disrupts the distal glial sealing in the RP retina. Confocal micrographs of vertical sections labeled with ß-tubulin (A-C) and GFAP (D-F) in saline treated RP (A, D) and AAA treated RP retinas after 3 days (B, E) and 2 weeks (C, F). The fibrotic processes were sealing the outer retina of saline-treated P33 RP retina (A, D, arrows). After 3 days of AAA post-injection, ß-tubulin and GFAP immunoreactive fibrotic sealing disappeared at the outer retina (B, E, arrowheads). After 2 weeks of AAA post-injection, the thick distal glial sealing labeled with ß-tubulin and GFAP reappeared in the outer retina (C, F, arrows). D, days; OPL, outer plexiform layer; INL, inner nuclear layer. Scale bar = 10 μm.

Sensitive cellular response markers associated with damage in retinal diseases are the up-regulation of cytoskeletal proteins, including glial fibrillary acidic protein (GFAP) [23–25] and ß-tubulin [26, 27]. A prominent example of the gliotic responses of Müller cells is correlated with their structural hypertrophy with increased expression of ß-tubulin and GFAP, which form distal fibrotic sealing in RP retinas [1, 2]. We hypothesized that the cones are redistributed with AAA due to its effects on distal glial sealing. To determine how the distal fibrotic processes of Müller cells responded to AAA treatment, we examined ß-tubulin and GFAP labeling in vertical sections of saline-treated and AAA-treated RP retinas. We examined the effects of AAA on distal fibrotic processes of Müller cells 3 days and 2 weeks following injection. In RP control retinas, we observed bundles of fibrotic processes of Müller cells labeled with ß-tubulin at the outer retina (Fig 2A, arrows). The fibrotic processes sealed the outer retina. After 3 days of AAA post-injection, we observed ß-tubulin immunoreactivity throughout the vertical section of the retina. However, we did not observe the ß-tubulin-immunoreactive fibrotic processes sealing the outer retina (Fig 2B, arrowheads). After 2 weeks of AAA post-injection, the thickened distal glial sealing labeled with ß-tubulin reappeared in the outer retina (Fig 2C, arrows). The distal fibrotic processes of Müller cells at the outer retina immunologically labeled by GFAP showed a similar pattern to ß-tubulin expression in vertical sections (Fig 2D–2F). These results suggest that AAA treatment transiently disrupted the distal fibrotic glial sealing at the outer retina.

### AAA transiently suppressed ZO-1 expression in the RP retina

**Fig 3:**
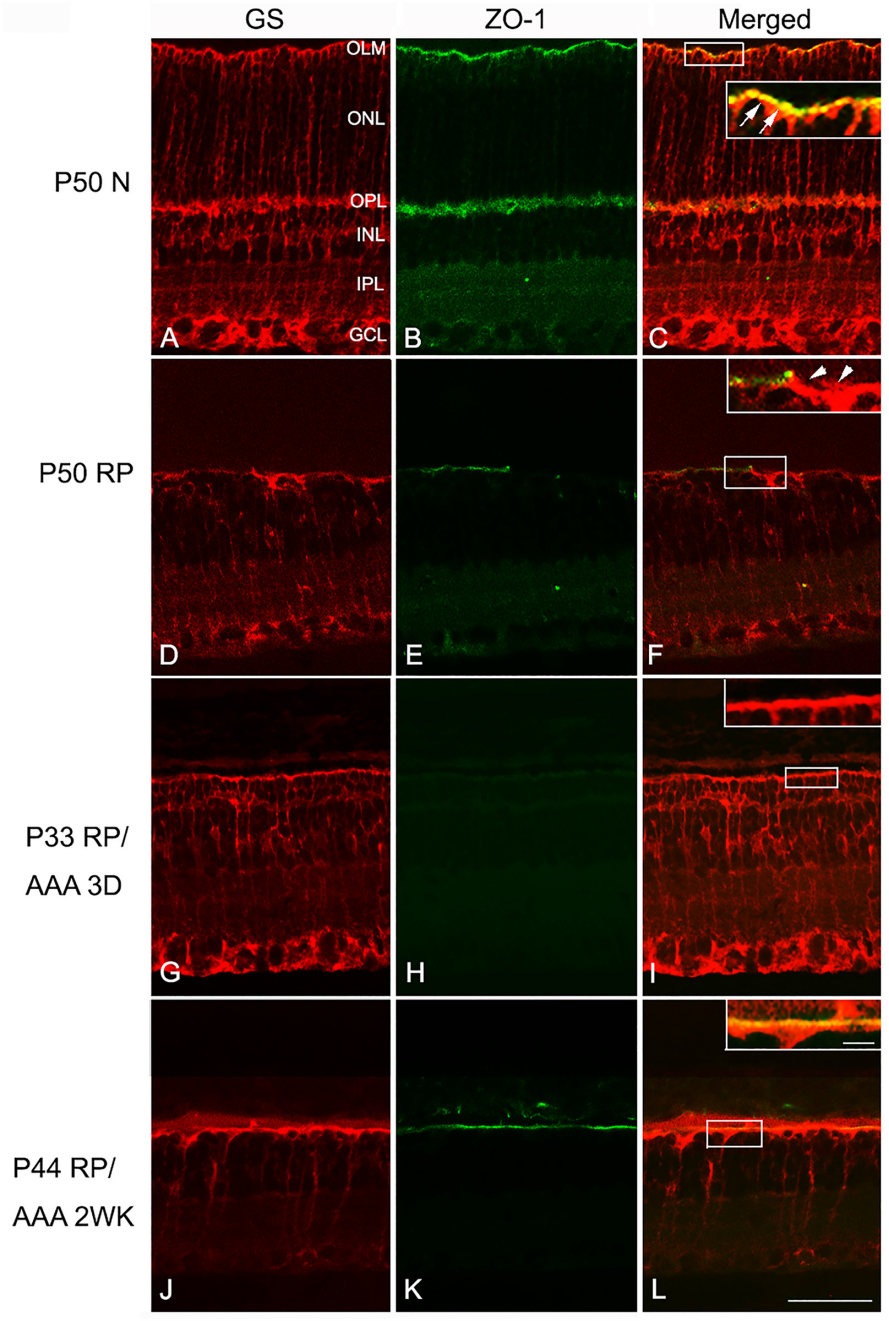
AAA transiently suppressed ZO-1 expression in the RP retina. Confocal micrographs of vertical saline-treated P50 normal (A-C), saline-treated P50 RP (D-F), AAA-treated P33 RP, and AAA-treated P44 RP retinal sections processed for GS (A, D, G, J) and ZO-1 (B, E, H, K) immunohistochemical staining patterns. Double exposure shows that the ZO-1 is expressed and co-localized with GS at the OLM (C inset—arrows). Double labeling of GS (D) and ZO-1 (E) in P50 RP retina shows that the ZO-1 expression is weaker and more fragmented (F inset—arrowheads) but still co-localized with GS at the OLM. In AAA-treated P33 RP retina (G-I), GS immunohistochemical staining still appears in Müller cells but ZO-1 is no longer expressed in outer part of the retina (I inset). In AAA-treated P44 RP retina, ZO-1 reappeared after 2 weeks of AAA treatment in RP retinas (K, L inset). AAA, DL-alpha-aminoadipic acid (AAA); N normal; D days; WK weeks; RP, Retinitis Pigmentosa; GS, glutamine synthetase; ZO-1, zonula occludens 1; OLM, outer limiting membrane; ONL, outer nuclear layer; OPL, outer plexiform layer; INL, inner nuclear layer; IPL, inner plexiform layer, GL, ganglion cell layer. Scale bar = 50 μm, 10 μm in inset.

AAA reversibly disrupts the integrity of outer limiting membrane (OLM) potentially by inducing toxicity in Müller cells [28, 29]. Also, ZO-1 expression at the OLM was shown to be discontinuous and fragmented during the drug application [9]. This specialized adheren-junction associated protein ZO-1 appears between the cones and the processes of Müller cells in the cone rings in RP retinas [7]. Thus, we explored the behavior of ZO-1 in RP after AAA treatment at P30 by examining ZO-1 expression after 3 (P33) days and 2 (P44) weeks. The retinas were labeled for GS, a marker of Müller cells (Fig 3, red) and ZO-1 (Fig 3, green). In normal retinas, Müller-cell processes were present throughout the retina (Fig 3A) and ZO-l immunological staining appeared at the OLM (Fig 3B). In the OLM region, ZO-1 was expressed and co-localized with GS (Fig 3C inset, arrows). More importantly, ZO-1 formed a continuous line at the OLM. In contrast, the RP retina showed discontinuous and fragmented immunological staining for ZO-1 at the OLM (Fig 3E). Double immunological labeling of GS (Fig 3D) and ZO-1 (Fig 3E) showed that the ZO-1 expression was weaker and more fragmented (Fig 3F inset, arrowheads) but still co-localized with GS at the outer part of the retina. Interestingly, AAA treatment did not affect the overall expression of GS (Fig 3G). The AAA treatment suppressed ZO-1 expression after 3 days (Fig 3H and 3I inset). However, we observed reappearance of ZO-1 (Fig 3K) at the outer retina 2 weeks after the AAA post-injection (Fig 3J–3L inset).

**Fig 4:**
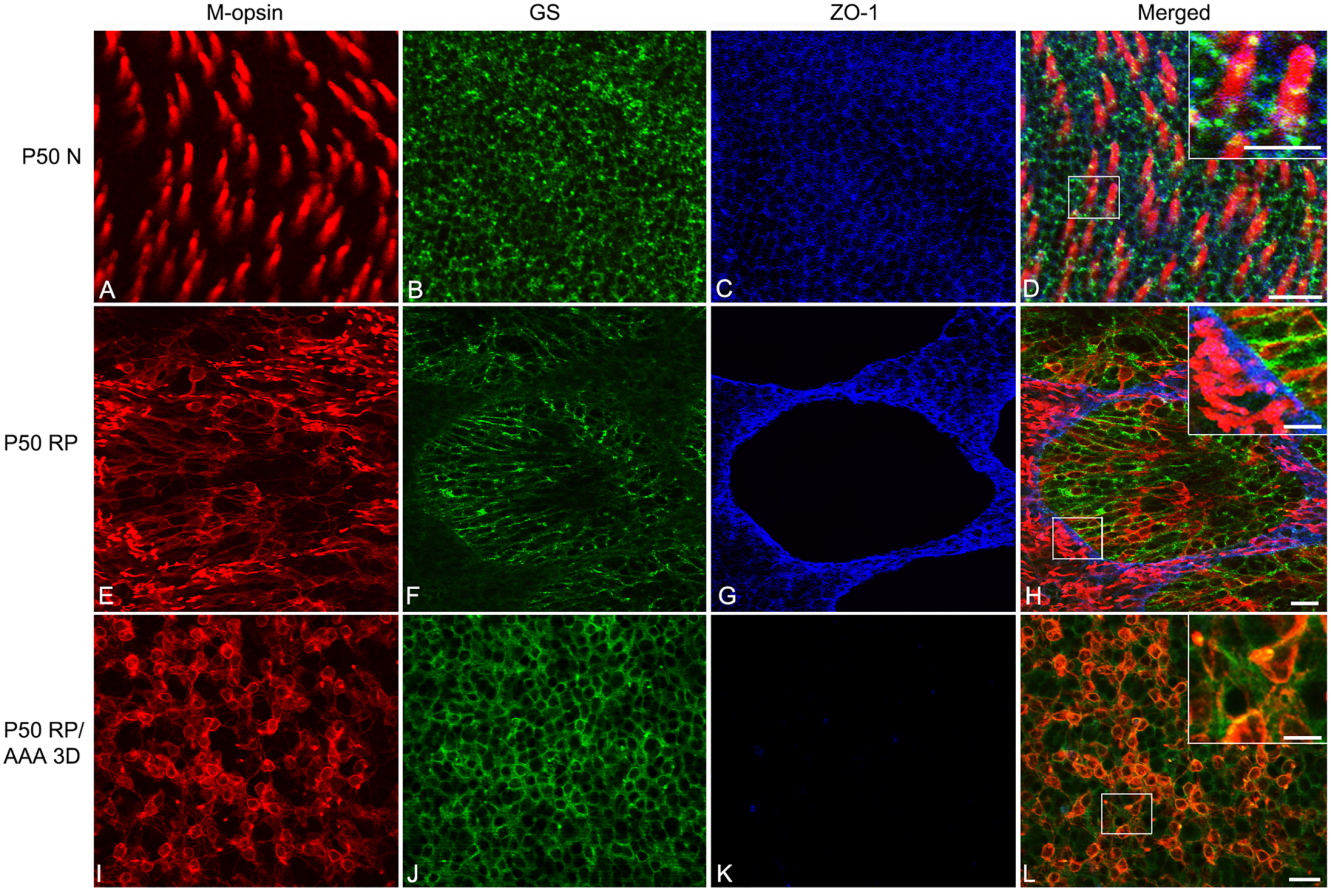
AAA disrupts cone rings by affecting ZO-1 expression between Müller cells and cones. Confocal micrographs taken from whole-mounts of saline-treated P50 normal (A-D), saline-treated P50 RP (E-H), and AAA-treated P50 RP (I-L) retinas processed for M-opsin (red), GS (green), and ZO-1 (blue) show immunohistochemical staining patterns. Saline and AAA (10ug/ml) were injected at P47. The micrographs show P50 N (A-D) retinas 3 days post-saline application. Triple immunohistochemical labeling of M-opsin (A), GS (B), and ZO-1 (C) shows that ZO-1 is closely associated between cone inner segments and the apical processes of Müller cells in P50 normal retinas (D inset). The micrographs for P50 RP (E-H) retinas show 3 days post-saline application. Triple labeling of M-opsin (E), GS (F), and ZO-1 (G) shows that the ZO-1 is closely associated with segments of photoreceptors and processes of Müller cells in rings (H inset). The micrographs for AAA-treated P50 RP retinas show 3 days post-application of the drug. Triple labeling of M-opsin (I), GS (J), and ZO-1 (K) shows that the ZO-1 is no longer expressed between cones and Müller cells with AAA treatment (L inset). In addition, Müller cell processes are homogenously distributed (J). AAA, DL-alpha-aminoadipic acid (AAA); N normal; D days; RP, Retinitis Pigmentosa; GS, glutamine synthetase; ZO-1, zonula occludens 1. Scale bar = 20 μm, 10 μm in inset.

Whole-mounts were triple labeled with antibodies against M-opsin, GS, and ZO-1. Fig 4 shows an example of P50 normal, P50 RP control, and AAA-treated P50 RP (after 3 days of AAA post-injection) retina whole-mounts processed for these antibodies. In this figure, the focal plane of the outer retina was examined. The normal retina treated with saline labeled M-opsin cones segments throughout the photoreceptor array (Fig 4A and 4D red). GS immunoreactivity displayed the normal spatially homogeneous mesh network of Müller cell processes (Fig 4B and 4D green). In the OLM region, ZO-1 was expressed (Fig 4C and 4D blue). We confirmed that triple immunohistochemical staining pattern with these markers in photoreceptor inner segments and the apical processes of Müller cells are closely associated with ZO-1 expression (Fig 4D inset). In RP control retinas, we again observed an array of cone rings (Fig 4E and 4H). Furthermore, we observed that the processes of Müller cells formed broccoli-like shapes (Fig 4F and 4H) and ZO-1 formed a network of rings (Fig 4G and 4H). The labeling showed that the ZO-1 also underwent remodeling, which allowed the Müller cells processes and cones to re-establish contact (Fig 4H inset). In contrast, RP eyes injected with AAA revealed neither rings of cones (Fig 4I) nor broccoli-like processes of Müller cells (Fig 4J). Müller-cell processes were homogeneously distributed with cones (Fig 4J and 4L). Furthermore, we also observed a disappearance of ZO-1 expression after 3 days of AAA treatment (Fig 4K), which coincided with the rearrangement of the cones (Fig 4K and 4L inset). Therefore, we propose that AAA disrupts cone rings by weakening the distal fibrotic processes of Müller cells (Fig 2) and also by affecting ZO-1 expression between Müller cells and cones (Figs 3 and 4).

### ZO-1 represents a critical component for cone rearrangement in RP retina

**Fig 5:**
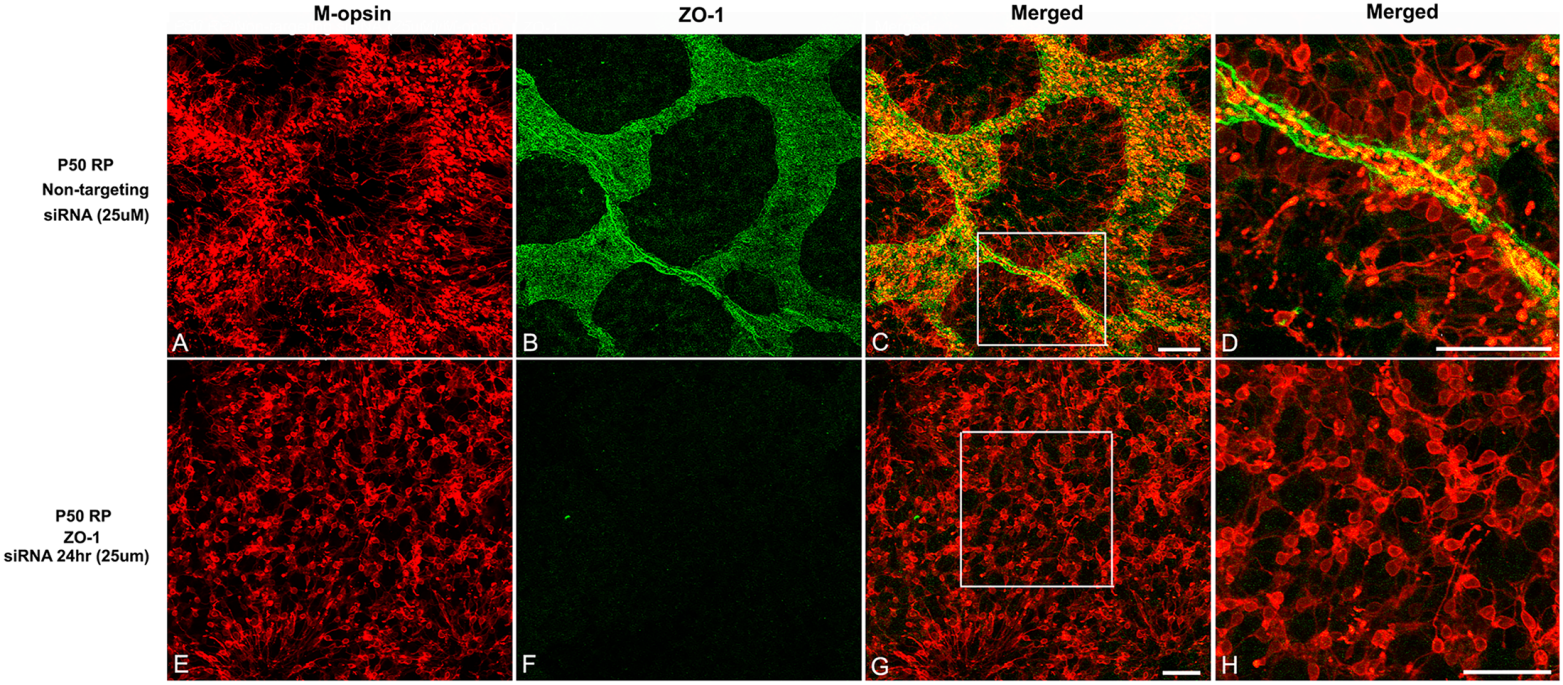
ZO-1 represents a critical component for cone rearrangement in RP retina. Whole-mounts for M-opsin (red) and ZO-1 (green) immunohistochemical staining in P50 RP retinas treated with non-targeted siRNA (A-D) and ZO-1 siRNA (25uM, E-H) for 24 hrs. In control retina, ZO-1 is closely associated with segments of photoreceptors (C, D). In ZO-1 siRNA treated retina, cones are re-occupying the space homogeneously (E, G) and ZO-1 is suppressed (F, G). D and H are higher-power micrographs of C, G, respectively. RP, Retinitis Pigmentosa; ZO-1, zonula occludens 1. Scale bar = 50μm.

To confirm if the down-regulation of ZO-1 is essential to disrupt the cone rings, we suppressed ZO-1 expression using ZO-1 siRNA technology. If ZO-1 siRNA leads to the disruption of cone rings then we propose that it plays a critical functional role in shaping the ring mosaic in RP. In RP retinas treated with the control, non-targeting siRNA for 24 hrs, we still observed an array of cones rings (Fig 5A). Furthermore, we observed ZO-1 expression in a network of rings (Fig 5B). Merged immunohistochemical images of M-opsin and ZO-1 showed that the segments of cones were closely associated with ZO-1 (Fig 5C and 5D). In ZO-1-siRNA-treated retina, cone rings were disrupted (Fig 5E) and ZO-1 expression was successfully suppressed (Fig 5F). Thus, the specific inhibition of ZO-1 promoted the disruption of cone rings in RP retina (Fig 5G and 5H).

**Fig 6:**
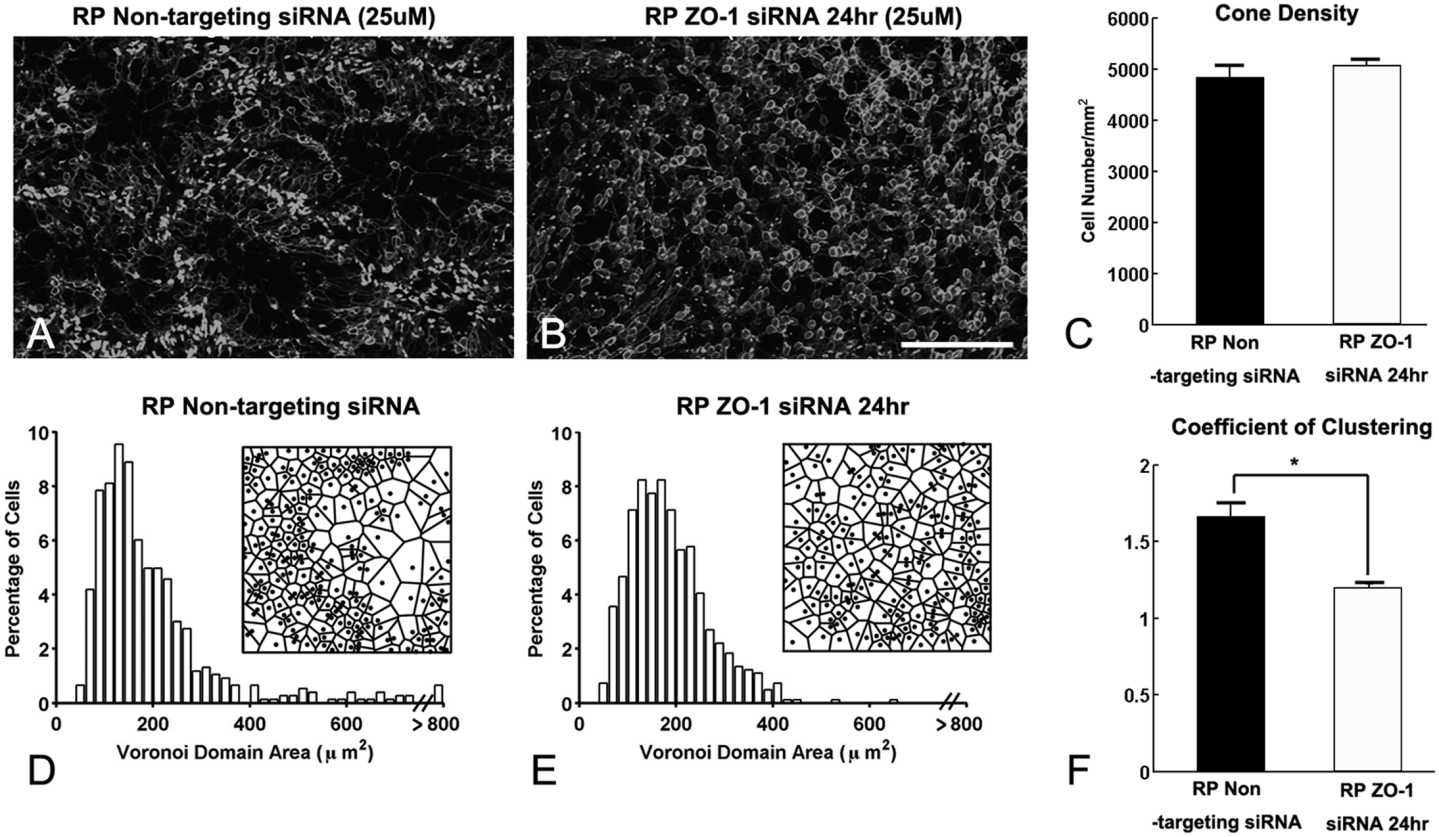
ZO-1 expression between cones and Müller cell processes are sufficient for the maintenance of rings. Confocal micrographs of whole-mounts processed for M-opsin immunohistochemical staining in non-targeted siRNA for 24 hr (A), ZO-1 siRNA treated for 24 hr (25uM, B) at P50. ZO-1 siRNA treated RP retinas show disruption of M-opsin cone rings in 24 hrs. The summary graph illustrates mean cone density (C) measured from the 1x1 mm^2^ sampling areas (for details, see methods) of non-targeted siRNA RP and ZO-1 siRNA treated RP retinas (n = 4 animals per group). The density of M-opsin cones in ZO-1 siRNA treated RP retina after 24 hrs showed no significant difference from non-targeted RP retinas. Histograms generated from the Voronoi analysis on the 1x1 mm^2^ sampling areas from non-targeted (D) and ZO-1 siRNA treated RP (E) retinas. Results are shown with survival times of 24 hrs. Examples (~ 170 μm x 170 μm) of the resulting Voronoi domains are shown for each group (D, E). The summary graphs for the mean skewness values obtained from the Voronoi domain distribution curves are plotted for each groups (D, E). Also, the graphs for the mean coefficient of clustering measured in all groups are illustrated (F). Data are presented as mean ± standard error. The symbol * indicates p < 0.05. ZO-1, zonula occludens 1; P, postnatal; D, day; RP, Retinitis Pigmentosa, Scale bar = 100 μm.

To further examine the effects of ZO-1 siRNA on the rearrangement of cones, we also performed Voronoi domain analysis on the cone mosaic. Consistent with the data in Fig 1, we observed ring-like pattern of cones in non-targeting siRNA RP retina (Fig 6A). In contrast, we observed disruption of cone rings after 24 hrs of ZO-1 siRNA in RP retina (Fig 6B). The cone density was not significantly different between the specific control, non-targeting siRNA, and ZO-1 siRNA treated RP retinas (Fig 6C, p = 0.3952). RP retinas treated with the control siRNA showed a long tail in the VD histogram (Fig 6D) and high CC, which indicate the existence of ring structures (Fig 6A). However, the number of larger VD domains decreased in ZO-1 siRNA treated RP retinas (Fig 6E). Moreover, the CC was significantly reduced in these retinas (Fig 6F, p = 0.0021). These dramatic and significant effects of ZO-1 siRNA indicate that ZO-1 expression between cones and Müller cells processes are sufficient for the maintenance of rings in the RP retina.

### ZO-1 is important for the formation of glial sealing in the outer retina

**Fig 7:**
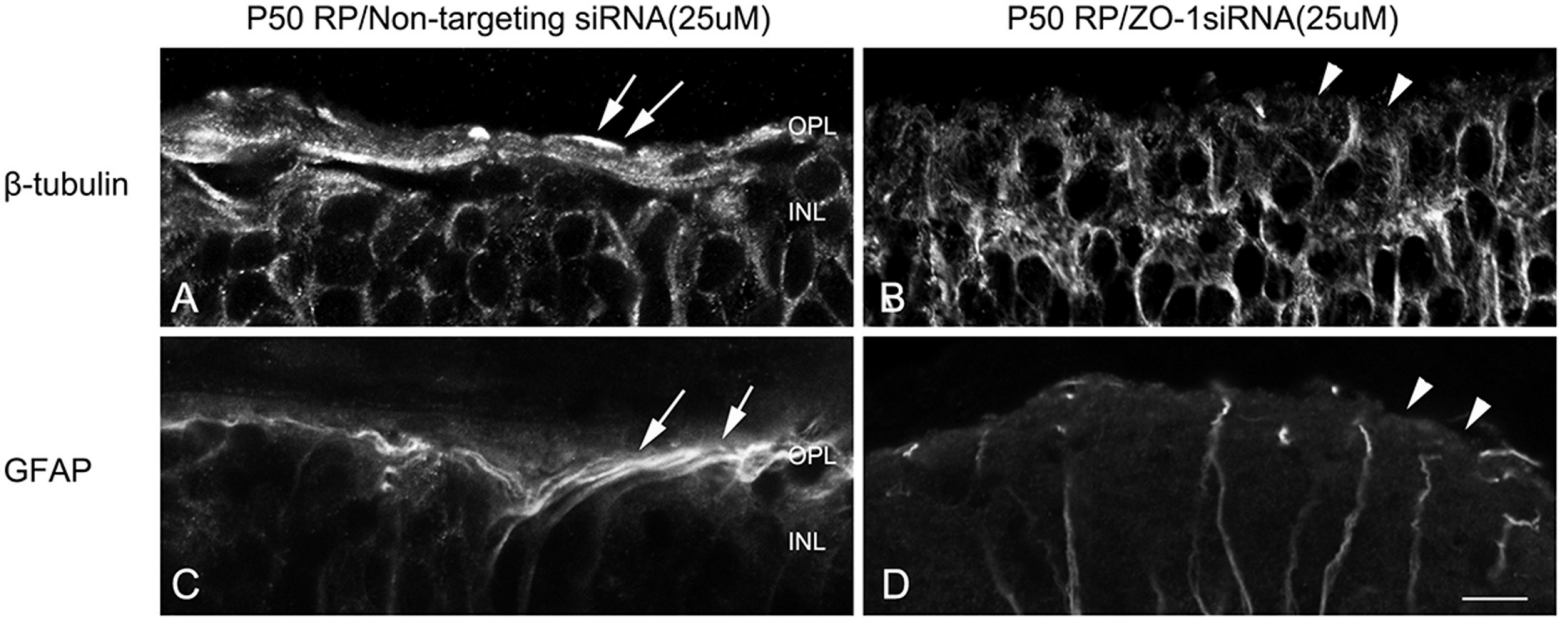
ZO-1 is important for the formation of glial sealing in the outer retina. Confocal micrographs of vertical sections labeled with ß-tubulin (A, B) and GFAP (C,D) in retinas treated with non-targeting siRNA (A, C) and ZO-1 siRNA (B, D). The fibrotic processes were labeled with ß-tubulin (A) and GFAP (C) at the outer retina of non-targeting siRNA treated RP retinas (A, C, arrows). After 3 days of AAA post-injection, ß-tubulin and GFAP immunoreactive fibrotic sealing disappeared at the outer retina (B, D, arrowheads). OPL, outer plexiform layer; INL, inner nuclear layer. Scale bar = 10 μm.

Since ZO-1 siRNA treatment disrupted cone rings in a similar manner as AAA treatment, we tested whether or not the distal glial sealing would also be disrupted by ZO-1 siRNA treatment. Similarly, we examined both ß-tubulin and GFAP immunological labeling in vertical sections of RP retinas treated with control siRNA and ZO-1 siRNA after 24 hrs (Fig 7). In RP retinas treated with control siRNA, we observed stronger ß-tubulin labeled fibrotic processes of Müller cells forming glial sealing at the outer retina (Fig 7A, arrows). After 24 hr ZO-1 siRNA post-injection, we did not observe glial sealing that was apparent in control retina (Fig 7B, arrowheads). GFAP immunoreactivity was present in bundles of fibrotic processes of Müller cells at the outer retinas of the control, non-targeting siRNA (Fig 7C, arrows). In contrast, we did not observe processes of Müller cells sealing the outer retina in ZO-1 siRNA treated retina (Fig 7D, arrowheads). These results suggested that ZO-1 is important for the formation of glial sealing in the outer retina.

## Discussion

### Dramatic effect of AAA treatment on the cone distribution and glial sealing

With a single intravitreal injection in RP retinas of AAA at P30 the rings of cones are disrupted without reducing the cone numbers at P33 (Fig 1). In addition, Müller cells processes were no longer clustered in broccoli-like shapes and the Müller cells processes were wrapping around the redistributed cones in the outer retina (Fig 4L). The Voronoi domain analysis confirmed disruption of cone rings statistically (Fig 1D–1F). These dramatic changes in cone distribution may be due to the changes in the integrity of the outer retina where Müller cells processes formed a dense fibrotic sealing (Fig 2 [6, 7]). This is supported by impairment of OLM integrity and presence of photoreceptor mislocalization at the outer retina after AAA treatment [9].

In S334ter-line-3 retina, we observed expression of GFAP and ß-tubulin throughout the Müller cells including fibrotic processes at the outer retina (Fig 2A and 2D). Furthermore, with the injection of AAA, the distal sealing formed by fibrotic processes of Müller cells was not apparent with ß-tubulin and GFAP after 3 days of post-injection (Fig 2B and 2E). This result is somewhat different from the previous study showing increased expression of cytoskeletal proteins including ß-tubulin and GFAP in disrupted Müller cells [29–31]. This discrepancy may be due to lower doses of AAA used in this study than those in other studies cited above and had limited effect on Müller cells toxicity. The thick distal glial sealing labeled with ß-tubulin and GFAP reappeared in the outer retina after 2 weeks of post-injection (Fig 2C). The transient effects of AAA on distal fibrotic glial sealing at the outer retina may be due, in part, to the disrupting adherens junctions at the outer retina. Normally, ZO-1 [10, 11, 13] is present between the inner segments of rods and cones and the apical processes of Müller cells [12]. In our previous study we showed that ZO-1 is expressed between the photoreceptor segments and the processes of Müller cells and also in between the Müller cells processes [7]. Upon injection of AAA, we observed a transient disappearance of ZO-1 expression after 3 days of AAA treatment (Figs 3 and 4); however, it reappeared 2 weeks after treatment (Fig 5). This disappearance coincided with cone mosaic rearrangement (Fig 2C). Therefore, we suggest that suppression of ZO-1 allows the rearrangement of cone mosaics and the reappearance of ZO-1 fixed the spatial distribution of cones. The support for this hypothesis comes from reversible effects of AAA on ZO-1 and the integrity of OLM [9]. Furthermore, pharmacological interference or genetic disruption of various components of the adherens junction and /or the supporting scaffold leads to significant impairment in the OLM integrity [32, 33]. In either case, AAA suppression of ZO-1 expression is still an unresolved mechanism. But since the junctional complexes at hetero-cellular adhesion sites are composed of many proteins that may traffic as supramolecular complexes [34], multiple pathways could effectively suppress ZO-1 expression.

### Effects of ZO-1 on cone distribution and glial sealing

In the RP retinas, ZO-1 expression still exists in the network of cone rings [7]. However, ZO-1 siRNA inhibition spreads cones out in the retina homogeneously without reducing cone number (Fig 5), which was confirmed statistically with Voronoi domain analysis (Fig 6). These results were mirrored in cone distribution in AAA-treated RP retina (Fig 1). These findings suggest that the ZO-1 represents at least one critical component for cone rearrangement in RP retina. Again, this dramatic change in cone distribution may be due to the changes in the integrity of the outer retina where Müller cells processes formed a dense fibrotic sealing (Fig 2 [6, 7]). ZO-1 is known to act as a molecular scaffold that organizes, assembles, and links the tight junctional complexes to the cytoskeleton through a number of protein-protein interactions[35]. Of note, there are significant differences in OLM adherens junction composition in the different models. In normal mice, these junctions form between photoreceptors and Müller cells. However, in another RP model, for example, *PDE6β*^*rd1/rd1*^, many junctions formed directly in between Müller cells, indicating significant OLM remodeling [36]. Thus suppressing the ZO-1 expression may weaken the bundles of filament by affecting the tight junctional complexes within them at the distal sealing. Therefore, in our study we have shown that ZO-1 is a critical component forming the glial sealing in the diseased retina.

### Therapeutic implications

The outer nuclear layer (ONL) of the vertebrate retina contains a tightly packed, uniform array of rods and cones, which is essential to ensure that the visual world is regularly sampled with no empty visual space. However, cones in the S334ter rat model of RP were recently shown both to survive for a longer period of time after the early rod deaths and to remodel in their mosaic pattern into orderly arrays of rings [4, 5, 7]. The relevance of cone rings extends beyond the S334ter RP model with well documented studies highlighting the appearance of dark patches or ‘‘holes of photoreceptors” in both animal models such as the cyclin D1 (cd1) mutant and P23H-line1 rat [37, 38], plus human retinal dystrophy, inherited retinal degeneration, and photo-pigment genetic perturbations in M-opsin cones [39–42]. Thus, the cones in ring, though frequently absent in RP, is a major pathologic hallmark of retinal dystrophic conditions. The center of these rings lack photoreceptors, indicating regional loss of visual function (Yu WQ, *et al*. *IOVS* 2014; 55: ARVO E-Abstract 6198). Therefore, investigation of the pathological etiology of cone rings is both fundamentally interesting and informative. Furthermore, knowledge on modulating and rearranging photoreceptors from the ring patterns into a more homogenous distribution may improve visual performance in these patients. In the current study, we clearly demonstrated for the first time that siRNA inhibition of ZO-1 expression spread cones out in the retina homogeneously leaving no empty space in RP and the dosage used for ZO-1 siRNA did not harm cones up to 24 hrs. However, additional studies are essential to determine if the effect on cone density can be sustained for a longer duration. Previous studies have shown that higher doses of ZO-1 siRNA can reduce ZO-1 expression, but results in photoreceptor death 48 hrs after treatment [12]. Furthermore, ZO-1 exists at the tight junctions of the retinal pigment epithelium [43] and at the composition of tight or adherens junctions around gap-junctional plaques at the outer plexiform layer [44]. Therefore, effects of ZO-1 siRNA need to be carefully monitored in order to translate its potential use into a pharmacological tool of preventing or slowing progressive forms of retinal dystrophy. Strong evidence exists that targeted disruption of the OLM junctional proteins enhances the integration and migration of photoreceptor transplantation in degenerating retina [12]. Therefore, developing siRNA technology on proteins that are involved in glial sealing may lead to substantial increases in transplanted cell survival in degenerating retinas.

## Conclusion

In our experimental RP rat model, AAA treatment reveals disruption of cone rings and distal fibrotic processes of Müller cells, in part, by suppression of ZO-1 expression. Furthermore, we discovered the importance of ZO-1’s role by silencing its expression through siRNA technology to preserve cone rings and to contribute significantly to glial sealing formation. This represents a key element to identifying the host of essential components involved in retinal degeneration’s etiology and potential therapeutic targets to prevent blindness.

## Supporting Information

S1 Fig Example of P33 RP nuclei position map. Nuclei positions map was constructed by marking the location of cell bodies using white dots. Applying white dot allowed easy identification of the position of each M-opsin positive cell in the retinal area. Also, using these images, Voronoi domain and the coefficient of clustering was measured.
(DOCX)

S1 Table The mean density and coefficient of clustering of M-opsin cones in saline–treated and AAA-treated RP retinas. The mean cone density was measured from the 1x1 mm^2^ sampling areas (for details, see methods) of saline-treated RP (P33 RP) and AAA-treated RP retinas (P33RP AAA 3D) (n = 3 animals per group). The mean coefficient of clustering was measured in all groups (Fig 1).
(DOCX)

S2 Table Cone coordinates of P33 RP retinas. The x and y are the coordinates of cones extracted from white-dot images. All the cone mosaic analyses are based on the coordinates.
(XLSX)

S3 Table Cone coordinates of P33 AAA 3D RP retinas. The x and y are the coordinates of cones extracted from white-dot images. All the cone mosaic analyses are based on the coordinates.
(XLSX)

S4 Table The mean density and coefficient of clustering of M-opsin cones in non-targeting siRNA-treated and ZO-1 siRNA-treated RP retinas. The mean cone density was measured from the 1x1 mm^2^ sampling areas (for details, see methods) of non-targeting ZO-1 siRNA-treated RP and ZO-1 siRNA-treated RP retinas (n = 4 animals per group). The mean coefficient of clustering was measured in all groups (Fig 6).
(DOCX)

S5 Table Cone coordinates of non-targeting ZO-1 siRNA RP retinas. The x and y are the coordinates of cones extracted from white-dot images. All the cone mosaic analyses are based on the coordinates.
(XLSX)

S6 Table Cone coordinates of ZO-1 siRNA RP retinas. The x and y are the coordinates of cones extracted from white-dot images. All the cone mosaic analyses are based on the coordinates.
(XLSX)
