# Supplementary material for: Reshaping the Cone-Mosaic in a Rat Model of Retinitis Pigmentosa: Modulatory Role of ZO-1 Expression in DL-Alpha-Aminoadipic Acid Reshaping
Source: PLoS One. 2016 Mar 15;11(3):e0151668. doi: 10.1371/journal.pone.0151668 (PMC4792433; doi:10.1371/journal.pone.0151668)
Supplement: S1 Table — The mean cone density was measured from the 1x1 mm2 sampling areas (for details, see methods) of saline-treated RP (P33 RP) and AAA-treated RP retinas (P33RP AAA 3D) (n = 3 animals per group). The mean coefficient of clustering was measured in all groups (Fig 1). (DOCX) [file pone.0151668.s002.docx]

| S1 Table |  |  |  |
| --- | --- | --- | --- |
|  | Sample 1 | Sample 2 | Sample 3 |
|  | cone density | cone density | cone density |
| P33 RP | 5748.002177 | 5479.03228 | 5572.31477 |
| P33 RP AAA 3D | 6046.910921 | 5113.3381 | 4574.47891 |
|  |  |  |  |
|  |  |  |  |
|  | Sample 1 | Sample 2 | Sample 3 |
|  | coefficient of clustering | coefficient of clustering | coefficient of clustering |
| P33 RP | 1.709876529 | 1.6159503 | 2.0757 |
| P33 RP AAA 3D | 1.402119078 | 1.28670572 | 1.27598113 |
