# Supplementary material for: Reshaping the Cone-Mosaic in a Rat Model of Retinitis Pigmentosa: Modulatory Role of ZO-1 Expression in DL-Alpha-Aminoadipic Acid Reshaping
Source: PLoS One. 2016 Mar 15;11(3):e0151668. doi: 10.1371/journal.pone.0151668 (PMC4792433; doi:10.1371/journal.pone.0151668)
Supplement: S4 Table — The mean cone density was measured from the 1x1 mm2 sampling areas (for details, see methods) of non-targeting ZO-1 siRNA-treated RP and ZO-1 siRNA-treated RP retinas (n = 4 animals per group). The mean coefficient of clustering was measured in all groups (Fig 6). (DOCX) [file pone.0151668.s005.docx]

S4 Table

|  | Sample 1 | Sample 2 | Sample 3 | Sample 4 |
| --- | --- | --- | --- | --- |
|  | cone density | cone density | cone density | cone density |
| non-targeting ZO-1 siRNA RP | 4431.818182 | 5394.15584 | 4507.79221 | 5039.61039 |
| ZO-1 siRNA RP | 4861.406553 | 4925 | 5375.10161 | 5150.09736 |
|  |  |  |  |  |
|  |  |  |  |  |
|  | Sample 1 | Sample 2 | Sample 3 | Sample 4 |
|  | coefficient of clustering | coefficient of clustering | coefficient of clustering | coefficient of clustering |
| non-targeting ZO-1 siRNA RP | 1.601087465 | 1.83229878 | 1.7681784 | 1.44997371 |
| ZO-1 siRNA RP | 1.217711018 | 1.25296642 | 1.14117556 | 1.19725164 |
